# Exploration and characterization of a newly isolated bacterium, Enterobacter quasihormaechei strain BDIFST24001, capable of producing rhamnolipid biosurfactant for oil remediation

**DOI:** 10.1099/acmi.0.000830.v4

**Published:** 2024-08-01

**Authors:** Afsana Habib Jui, Mohammad Nazrul Islam Bhuiyan, Banasree Bhowmik, Nazia Khatun, Abhijit Chowdhury, Riyadh Hossen Bhuiyan, Md. Alamgir Kabir, Md. Mamunur Rashid, Md. Habibur Rahman Bhuiyan, Sadia Afrin

**Affiliations:** 1 Institute of Food Science and Technology (IFST), Bangladesh Council of Scientific and Industrial Research (BCSIR), Dr. Qudrat-i-Khuda Road, Dhaka 1205, Bangladesh; 2 BCSIR Dhaka Laboratories, Bangladesh Council of Scientific and Industrial Research (BCSIR), Dr. Qudrat-i-Khuda Road, Dhaka 1205, Bangladesh

**Keywords:** biosurfactants, environmental pollution, *Enterobacter quasihormaechei*, oil remediation, rhamnolipids

## Abstract

Biosurfactants are naturally occurring compounds synthesized by micro-organisms that increasingly attract attention due to both their living area and application in various industries. In this study, we explore and characterize a novel bacterium, *Enterobacter quasihormaechei* strain BDIFST24001, isolated for its ability to produce rhamnolipid biosurfactants, with the aim of facilitating oil remediation processes. The isolation of this bacterium was carried out using Luria-Bertani (LB) broth media from environmental samples collected from oil-contaminated sites in Dhaka City. Screening tests, including the oil spreading method and drop collapse assay, were conducted to identify potential biosurfactant-producing strains, leading to the selection of *E. quasihormaechei* strain BDIFST24001 based on its favourable performance. Subsequent molecular identification revealed a high similarity of the strain’s 16S rRNA gene to *E. quasihormaechei*, which was corroborated through phylogenetic analysis. Further analysis of the biosurfactant produced by this strain indicated its rhamnolipid nature, as confirmed by FT-IR spectroscopy. The rhamnolipids exhibited promising surface-active properties, including a significant reduction in surface tension and emulsification activity, as evidenced by surface tension measurements and emulsification index assays. Optimization studies revealed that the optimal conditions for rhamnolipid production by *E. quasihormaechei* strain BDIFST24001 were a temperature of 37 °C, pH 10.0 and salinity of 4 %. The rhamnolipids produced by this strain demonstrated effective oil remediation capabilities, as observed through controlled experiments using petrol oil. The rhamnolipids effectively reduced the surface tension of the oil–water interface, facilitating the dispersion and emulsification of the oil phase in water. Overall, our findings highlight the potential of *E. quasihormaechei* strain BDIFST24001 as a promising candidate for biosurfactant-mediated oil spill cleanup and environmental remediation efforts.

**Impact Statement**

Discovery and description of *E. quasihormaechei* strain BDIFST24001 as a potential rhamnolipid biosurfactant producer have enormous progress on eco-friendly biotechnology in cleaning oil spill and environmental bioremediation. Indeed, such study not only indicates the ability of this new strain to produce rhamnolipids efficiently but also accords that biosurfactant successfully reduces the surface tension and promotes the emulsification of oil, which is a critical point of improvements of oil biodegradation. Moreover, the optimal conditions for rhamnolipids by *E. quasihormaechei* strain BDIFST24001 for rhamnolipid production can be considered with valuable scientific meaning for scaling biosurfactant production for practical environmental use. These developments confirm the assumption that this strain and the produced biosurfactants could be used as a potential producer in the future for oil pollution and the basis for green technology applications in environmental sustainability.

## Data Summary

The NCBI GenBank sequence of *E. quasihormaechei* strain BDIFST24001 used in this study has been deposited in GenBank under the accession number PP301330. The gene sequence is publicly available in GenBank.

## Introduction

In the realm of environmental sustainability and bioremediation, biosurfactants have emerged as valuable tools due to their eco-friendly nature and versatile applications. Derived from microbial sources, particularly bacteria, biosurfactants exhibit unique surface-active properties that enable them to reduce interfacial tension and enhance the solubilization and emulsification of hydrophobic compounds, making them promising agents for oil spill remediation. The increasing frequency of oil spills in marine environments is a serious threat to biodiversity and ecosystems. According to the Statista Research Department report, between 2010 and 2019, there was an average of 1.8 significant oil spills due to tanker incidents annually. Over 700 metric tons of oil was reported to have leaked in four separate oil spills in 2022 [1]. These incidents highlight the ongoing risk and environmental impact of oil spills. Despite efforts to improve safety measures and regulations, the frequency and magnitude of such accidents remain a concern. According to the International Tanker Owners Federation (ITOPF), between 1970 and 2019, approximately 5.86 million tons of oil was spilled into marine environments due to tanker transportation alone [1]. Every year, transoceanic shipping carries about 35 million barrels of petroleum, and there are many ship accidents, most of which result in a significant spill of petroleum into marine environments. Large-scale, toxic oil spills typically occur at marine sites. Spills and leaks of crude oil have the potential to cause terrible harm to aquatic environments [2]. There is a possibility that these hazardous substances will enter the marine environment during the extraction, transportation and refining of crude oil, which would have negative effects on all biotic life [3]. Conventional techniques for cleaning up oil spills involve the use of mechanical tools like oil booms and skimmers, but these are labor- and money-intensive procedures [1]. Chemicals can be used as oxidants, reductants, polymers, or precipitants in chemical remediation processes [4]. Chemical surfactants, on the other hand, have toxic environmental effects. Biosurfactants are surface active biomolecules produced by microbial (bacteria, yeast and fungi) metabolism, containing both hydrophilic group and lipophilic group [5]. Evaluable biotechnology technique known as ‘bioremediation’ is the process of using micro-organisms to mitigate and remove pollutants from a projected contaminated environment [6]. Because biosurfactants are more environmentally friendly, less toxic, biodegradable and have lower critical micelle concentration (CMC) values than chemical surfactants, they can be used as substitutes for chemical surfactants [7]. The critical micelle concentration refers to the minimum amount of surfactant required to achieve the lowest surface tension [8]. Biosurfactants are a type of surface-active compounds produced by micro-organisms, including bacteria, yeast and fungi. Biosurfactants are amphiphilic compounds that contain both hydrophilic (polar head) and hydrophobic (non-polar tail) domains. Surface-active compounds are categorized based on their molecular weight. Low-molecular-weight (LMW) surfactants lower the surface tension between two immiscible liquids, and high-molecular-weight (HMW) emulsifiers allow water-in-oil or oil-in-water emulsions [9,10]. The effectiveness of a surfactant is based on the extent to which it can lower surface tension. A highly effective biosurfactant can decrease the surface tension of water from 72 to below 35 mN/m [10]. Low molecular weight glycolipids (rhamnolipids, sophorolipids, etc.) are among the most widely used biosurfactants, and they have been thoroughly and extensively studied for their use in biotechnological applications. A glycolipid is a compound comprising a carbohydrate section and fatty acid chains connected by a glycosidic bond, acting as hydrophilic and hydrophobic links, respectively [11]. Biosurfactants can be produced by a number of bacterial species, including *Bacillus*, *Pseudomonas* and *Enterobacter*. Rooney *et al*. (2009) [12] reported the isolation of *E. hormaechei* from the biodiesel-contaminated soil, which was able to produce rhamnolipid-type biosurfactant [12]. Some other studies also reported the production of biosurfactants from *Enterobacter* spp. [13]. The delve of this study is to explore and characterize a newly isolated bacterium capable of producing biosurfactants, with the goal of assessing its potential for use in oil remediation processes.

## Methods

### Chemicals, reagents and culture media

Microbiological media were sourced from Hi-Media, India, while biochemical reagents and other chemicals were obtained from Sigma, USA. Additionally, all crude oil samples (including petrol, kerosene and diesel) were procured from the local market in Dhaka City, Bangladesh.

#### Sample collection and isolation of isolates

Samples were collected from various fuel stations in Dhaka City using sterile Ziploc bags. The oil-contaminated soil samples were promptly stored at 4 °C to maintain the integrity of the microbial consortium until further use. The serial dilution method was employed to isolate bacteria from the samples. For obtaining pure cultures, bacterial isolates were subsequently sub-cultured on Luria-Bertani (LB) agar plates. Stock cultures of pure isolates were prepared in 20% glycerol solution and stored at −20 °C for long-term preservation.

### Biochemical characterization of bacteria

The selected isolate underwent a series of morphological, cultural and biochemical tests, including Gram staining, oxidase test, catalase test, methyl red (MR) test, Voges–Proskauer (VP) test, citrate utilization test and motility test, following the protocols outlined in Bergey’s Manual of Determinative Bacteriology [14]. The Gram staining procedure begins by placing a drop of sterile water onto a sterile glass slide. A colony from a fresh bacterial culture is then smeared across the slide, dried and fixed. Crystal violet dye is applied to the smear for 1 min before being gently rinsed off with water. Following this, Gram’s iodine is added, then washed off and the slide is briefly treated with 70 % ethanol before immediate rinsing. Safranin dye is subsequently applied for 45 s, then rinsed off and the slide is left to dry completely before examination under a microscope. For the oxidase test, a filter paper is saturated with tetramethyl-*p*-phenylenediamine dihydrochloride and smeared with fresh bacterial culture. A colour change to indophenol blue within 10–30 s indicates the presence of cytochrome c oxidase, which oxidizes the reagent. In the catalase test, a small sample of a bacterial isolate is introduced into a 3% hydrogen peroxide solution. The rapid release of oxygen bubbles signifies the presence of catalase, while the absence of bubbles indicates a catalase-negative result. The methyl red (MR) test involves cultivating the test bacteria in a glucose-containing broth medium. The production of stable acids such as lactic, acetic, or formic acid by the bacteria results in a colour change of the methyl red indicator from yellow to red. For the Voges-Proskauer (VP) test, colonies from a 24-h-old culture are inoculated into broth and incubated aerobically for 18 to 24 h at 37 ± 2 °C. After incubation, 2 ml of broth is transferred to a sterile test tube, treated with 5 % α-naphthol solution (reagent A) and mixed. Then, 40 % KOH solution (reagent B) is added and mixed. In the presence of atmospheric oxygen and potassium hydroxide, acetoin is converted to diacetyl, which forms a red complex in the presence of alpha-naphthol and creatine. A pink to red colour at the medium’s surface indicates a positive VP result, while the absence of colour indicates a negative result. For the citrate utilization test, a vial containing Simmons citrate agar is inoculated with the test organism and incubated for 24–48 h at 37 °C. A positive result is indicated by a pH increase in the medium, changing the bromothymol blue indicator from green to blue. In the motility test, a test tube containing a semi-solid medium is inoculated with the test organism by stabbing. The tubes are incubated at 37 °C for 24–48 h. A positive result for motility is indicated by the diffusion of the organism away from the stab line, creating a turbidity or cloudiness throughout the medium. If the organism is non-motile, growth will only occur along the line of inoculation, leaving the rest of the medium clear. These tests, among others, are part of the IMViC series, which stands for indole, methyl red, Voges-Proskauer and citrate tests. They are commonly used to differentiate among members of the Enterobacteriaceae family, which includes a variety of pathogens such as *E. coli*, *Salmonella* and *Shigella*. Each test targets a specific metabolic capability that can help in identifying bacteria at the genus or species level.

### Molecular identification and phylogenetic tree construction

The bacterial DNA from the test isolate (B24) was extracted using a modified boiling method [15]. The isolates were grown in Luria-Bertani (LB) broth at the optimum temperature. After 24 h, 1 ml of the broth culture was transferred into an Eppendorf tube and centrifuged for 10 min at 10  000 r.p.m. The resulting pellet was collected, and 100 µl of RNase-free water was added and mixed thoroughly by vortexing. The Eppendorf tube was then boiled at 100 °C in a water bath for 5 min, followed by immediate placement in ice for another 10 min. Subsequently, 0.7 vol. of cold absolute ethanol was added to the supernatant containing DNA, and the mixture was centrifuged for 20 min. The upper aqueous phase was removed, and the genomic DNA was precipitated as a pellet by ethanol, which was washed with cold 70 % ethanol through further centrifugation. Finally, the DNA-containing pellet was air-dried and resuspended in Tris-ethylenediamine tetraacetic acid buffer (10 mM Tris-Hydrochloric acid, 1 mM Ethylenediamine tetraacetic acid and pH 8.0), and the DNA concentration was measured using a NanoDrop Spectrophotometer (Thermo Scientific). The bacterial DNA was then stored at −20 °C. To identify the biosurfactant-producing isolate (B24), 16S rRNA sequencing was performed with universal primers 27F: (5′-AGAGTTTGATCCTGGCTCAG-3′) and 1492R: (5′-TACGGTTACCTTGTTACGACTT-3′). PCR amplification involved denaturation at 94 °C for 5 min, followed by 35 cycles of denaturation at 94 °C for 1.5 min, annealing at 56 °C for 1 min and elongation at 72 °C for 1.5 min, with a final extension at 72 °C for 10 min. The PCR products were analysed via 1.5 % agarose gel electrophoresis and visualized using a gel documentation system (Thermo Scientific, USA). Sequences were aligned with those in the NCBI database by using ClustalW alignment method, and a phylogenetic tree was constructed using mega 11 software with the neighbor-joining DNA distance algorithm method.

### Screening for biosurfactant production

Luria-Bertani (LB) broth served as the seed medium for biosurfactant production studies. The culture was incubated in a rotary incubator at 120 r.p.m. for 24 h, maintaining a temperature of 37 °C. A 2 % (v/v) seed culture was inoculated into broth and incubated on a rotary shaker at 120 r.p.m. at 37 °C for an additional 24 h. Subsequently, the isolates were cultured in liquid minimal salt medium (MSM) containing glucose (5%) and crude oil (2%) as carbon sources, supplemented with Na_2_HPO_4_ (6 g/l), KH_2_PO_4_ (3 g/l), NaCl (0.5 g/l), NH_4_Cl (1 g/l) and MgSO_4_.7H_2_O (0.24 g/l). The cultures were then incubated for 3 days at 37 °C on a rotary shaker set at 120 r.p.m. After incubation, the cultures were centrifuged at 9000 r.p.m. for 10 min to obtain cell-free supernatant, which was used for preliminary testing of biosurfactant activity [16].

### Oil spreading test

In the oil spreading test, a Petri dish with a diameter of 90 mm was filled with approximately 20 ml of distilled water. A thin film (10 ml) of crude oil was created on the water’s surface. Subsequently, 200 µl of cell-free supernatant was delicately placed in the middle of the oily layer. After 30 s, the diameter of the area covered by the oil spreading was measured. Distilled water served as the negative control, while sodium dodecyl sulphate (SDS) was used as the positive control [17,18].

### Drop collapse test

The qualitative drop collapse test was conducted following the protocol outlined by Bodour and Miller-Maier [19]. In each well of a 96-well microtitre plate, 2 µl of petrol oil was dispensed. Following a 1 h equilibration period at 37 °C, 5 µl of supernatant was introduced onto the oil surface. The shape of the drop on the oil surface was observed after 1 min. When distilled water served as the control, culture supernatants causing the oil drop to collapse were identified as positive results, while drops that remained beaded were considered negative [20].

### Emulsification activity

To evaluate emulsification activity, 2 ml of cell-free supernatant was combined with 2 ml of crude oil (a blend of petrol, kerosene and diesel) and vortexed for 2 min. The mixture was left to stand for 24 h at room temperature before measurement. The emulsification index (E24) was determined by dividing the height of the emulsified layer by the total height of the liquid column and multiplying by 100 [21].

$Emulsification\;activity(\%)=\frac{Height\;of\;emulsion\;layer}{Total\;height}\times 100$

### Surface tension measurement

Surface tension measurements were conducted using a surface tensiometer (Cambridge Instrument Co. Ltd, UK), operating on the Du Nouy ring method principle [22]. The instrument was calibrated using distilled water (with a surface tension of 70 mN/m), while fresh MSM media acted as the negative control. Three measurements were taken, and the average value was calculated.

### Bacterial adhesion to hydrocarbon assay

The bacterial adhesion to hydrocarbon (BATH) assay was performed following the method described by Zargar *et al.* [23] with minor modifications [23]. A flask containing 100 ml of Luria broth (10.0 g/l tryptone, 5.0 g/l yeast extract, 5.0 g/l sodium chloride) was inoculated with 1 % of an overnight-grown seed culture, containing 18×10^8^ c.f.u. ml^−1^. The flasks were then incubated at 37 °C with constant shaking at 150 r.p.m. for 3 days until reaching the logarithmic phase. Subsequently, the cells were harvested by centrifugation at 9000 r.p.m. at 4 °C for 30 min. The harvested cells were resuspended in 25 ml of phosphate urea magnesium (PUM) sulphate buffer, containing K_2_HPO_4_ (16.948 g/l), urea (1.8 g/l), KH_2_PO_4_ (7.26 g/l) and MgSO_4_·7H_2_O (0.2 g/l). In a test tube, 6 ml of bacterial suspension and 3 ml of petrol were combined. The test tube was then pre-incubated at 37 °C for 10 min, followed by vortexing for 5 min and allowing 60 min for phase separation. The optical density (OD) of the aqueous phase before (OD_a_) and after petrol treatment (OD_b_) was measured at 400 nm using a UV-visible spectrophotometer (PG Instruments, USA). The percentage of bacterial cells adhering to petrol compared to the control (suspension without petrol addition) was calculated using the following formula:

%BATH = (1−(OD_b_/OD_a_)) × 100

### Optimization of the growth conditions of isolates

The organisms were cultured in minimal salt medium (MSM) under different conditions of temperature, pH and salinity to assess their impact on isolate growth for biosurfactant production. Growth patterns were monitored by measuring absorbance (OD) at 600 nm using a spectrophotometer at various time intervals.

### Biosurfactant production, extraction and purification

The acid precipitation method followed by solvent extraction (chloroform: methanol) was utilized for the extraction of crude biosurfactant [17]. A fresh culture of the chosen bacteria (28×10^8^ c.f.u. ml^−1^, OD 1.963 at 600 nm) was introduced into a flask containing 1 l of MSM (pH 11), supplemented with 2 % petrol oil and 5 % glucose as carbon sources. The flask was then incubated in a shaker incubator at 37 °C and 120 r.p.m. for 3 days. Following the incubation period, the bacterial culture supernatant was obtained by centrifugation at 10 000 r.p.m. for 20 min at 4 °C. The pH of the collected supernatant was adjusted to two and kept at 4 °C overnight. Subsequently, an equal volume of chloroform: methanol (2 : 1 v/v) was added at room temperature and thoroughly mixed. The formation of a white precipitate indicated the presence of biosurfactant. A separation funnel was employed to separate the organic phase (extract) from the aqueous phase (solvent). The emulsifier layer was then concentrated using a rotary evaporator (LabTech, USA) at 40 °C, resulting in a viscous brown-coloured product, which represented comparatively pure biosurfactant after the solvents were evaporated. Following that, the biosurfactant was further purified from crude extracts using silica gel (60 mesh) column chromatography. A solvent mixture of methanol (MeOH) and water (50 : 50 v/v) was employed for this purification process. The purified biosurfactant was then subjected to rotary evaporation, freeze-dried and stored at room temperature.

### UV spectroscopy

The absorbance spectra of a compound in solution or as a solid can be determined using ultraviolet-visible (UV-vis) spectroscopy. An analytical method that is frequently used to determine the amount of chemicals in a solution is the UV spectrophotometer (Analytic Specord, Germany). The wavelength of light emitted determines the chemical’s reflective qualities. By using UV-visible analysis with a scanning interval of 100 mm, the extracted biosurfactant was further characterized in the 200–800 nm range [24].

### Fourier-transform infrared spectroscopy

Fourier-transform infrared (FT-IR) spectroscopy analysis was conducted using a PerkinElmer Fourier-transform infrared spectrometer with a diamond-attenuated total reflection accessory (Frontier, PerkinElmer, UK; Software: Spectrum version 10.4.4). This technique enabled the detection of different functional groups. Spectral data were collected within the range of 650–4000 cm^−1^ at a resolution of 4 cm^−1^.

### Gas chromatography-Flame ionization detector (GC-FID) analysis

Fatty acid compositions of extracted biosurfactants were evaluated as their methyl esters with a slightly modified method of the American Oil Chemists’ Society official method ch 1–91 [25]. In brief, 20 mg of the extract was dissolved in 2 ml of acetone in a sealed centrifuge tube. Subsequently, 50 µl of 2 M methanolic KOH was added, and the mixture was vigorously shaken for 30 s. Following this, 2 ml of saturated Nacl was introduced, and the upper layer was passed through an anhydrous sodium sulphate column and collected in a wide-mouth 2 ml vial for GC analysis. The fatty acid composition was analysed using a GC-FID (Trace 1300, Thermo Scientific, PA, USA) equipped with a flame ionization detector and a fused silica capillary column (TR-FAME, 30 m × 0.25 mm × 0.25 µm, film thickness). The split injection technique (20 : 1) was used, with nitrogen serving as the carrier gas at a constant flow rate of 1 ml min^−1^. The injector temperature was set at 250 °C, while the starting oven temperature of 150 °C was maintained for 5 min. Subsequently, the temperature was increased to 200 °C at a rate of 5 °C min^−1^ and then to 240 °C for 5 min at a rate of 10 °C min^−1^. Identification of fatty acids was accomplished using respective fatty acid methyl ester standards (Supelco 37, USA), and the results were presented as relative percentages using the automated GC software (Chromeleon, version 7.00).

### Critical micelle concentration

The concentration at which surface tension is at its lowest and micelles start forming is known as a biosurfactant’s critical micelle concentration (CMC). Dried extracted biosurfactant was dissolved at different concentrations ranging from 1 to 500 mg l^−1^ in distilled water, and the changes in surface tension values were measured using Du Nouy ring method in order to assess the CMC of the biosurfactant.

### Oil spreading test

Extracted crude biosurfactant was used to test the oil-spreading ability on water surfaces. On the surface of the water in the Petri dish, 50 µl of petrol oil was applied until it formed a thin film. The biosurfactant was then added to the oil on the water’s surface and observed after 1–2 min to see how it affected the spreading of the oil.

### Investigating the mechanism of action of the isolated strain in the environment under varying biosurfactant concentrations

To assess the response of the isolated strain to its environment, we exposed it to five different concentrations of biosurfactants (10, 20, 50, 100 and 200 µg) over a 48 h period. Each experiment consisted of the growth media, the isolated strain and one of the specified concentrations of biosurfactants. Positive controls included both the growth media and the isolated strain, while a negative control comprised only the growth media. Optical density (OD) measurements were taken at 600 nm after 24 and 48 h to monitor growth and evaluate the impact of the biosurfactant concentrations on the strain’s behaviour.

### Statistical analysis

The experiments were conducted three times independently. The average value of these trials and their standard errors were computed using Microsoft Office Excel 2019.

## Results and discussion

### Isolation of biosurfactant-producing isolate

The biosurfactant-producing isolate B24 was isolated from environmental samples gathered from oil-contaminated sites through the utilization of LB agar media. In this particular study, a total of 25 bacterial isolates were isolated from various oil-contaminated samples. Each of these isolates underwent preliminary screening for biosurfactant production, involving a series of qualitative and quantitative tests. Rapid techniques, such as the oil spreading and drop collapse assays, were employed to identify microbial biosurfactant producers. Results from the oil spreading and drop collapse tests indicated that among the 25 isolates, 10 exhibited favourable outcomes in both assays (Table 1). The diameter of the test solution served as an indicator of higher activity and concentration, as recognized in previous studies [17]. Conversely, the oil spreading test was utilized to identify strains with lower activity and quantity of biosurfactants [26]. The drop collapse test relies on the destabilization of liquid droplets by surfactants. If the droplets of the supernatant contain biosurfactants, the supernatant will display a hydrophobic surface and lower interface tension, thereby resulting in broader-shaped droplets [27].

**Table 1.** Screening of bacterial isolates for biosurfactant production

Isolates no.	Oil spreading test			Drop collapse test		
	Petrol oil	Kerosene	Diesel	Petrol oil	Kerosene	Diesel
B2	5.0±0.52	3.5±0.29	3.8±0.17	**+**	**+**	**+**
B4	5.2±0.71	2.7±0.33	4.2±0.44	**+**	**+**	**+**
B9	5.0±0.51	5.3±0.89	5.2±0.17	**+**	**+**	**+**
B12	5.5±0.33	5.2±0.44	4.2±0.17	**+**	**+**	**+**
B18	5.0±0.34	4.2±0.17	3.8±0.44	**+**	**+**	**+**
B20	4.5±0.33	4.2±0.44	5.5±0.29	**+**	**+**	**+**
B22	4.9±0.42	5.5±0.76	5.7±0.44	**+**	**+**	**+**
B23	5.5±0.61	3.8±0.17	4.5±0.29	**+**	**+**	**+**
**B24**	**6.0±0.84**	**5.7±0.29**	**5.5±0.29**	**+**	**+**	**+**
B25	3.6±0.33	2.8±0.17	4.2±0.44	**+**	**+**	**+**

*All the Vvalues reported are averages of three replicates ± the standard error.

**Fig. 1.**
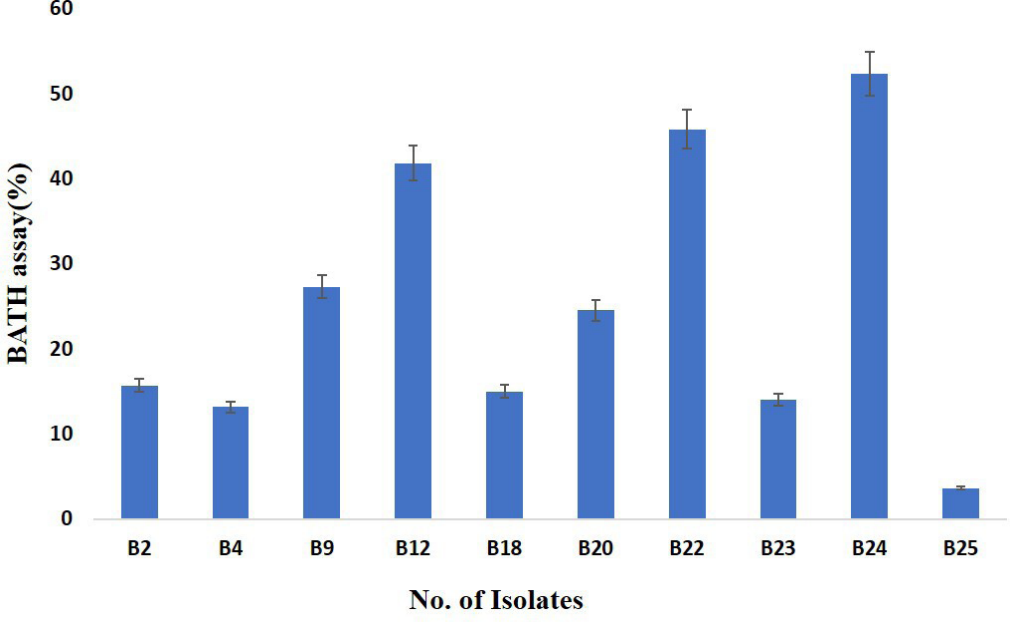
Bacterial adhesion to hydrocarbon test of isolates.

In BATH assay, bacteria undergo indirect screening for biosurfactant production through their adherence to hydrophobic compounds like crude oil. Utilizing petrol as a carbon source in biosurfactant production allows for its application as a hydrocarbon in this screening test. When cells adhere to oil droplets, they typically produce surface-active biosurfactants [28]. The range of cell adherence measured with crude oil for the isolates that tested positive varied from 3.20 to 52.27%. Notably, results highlighted that among the bacterial isolates screened, those designated as B24 exhibited the highest cell adherence with crude oil (petrol oil), as depicted in Fig. 1. Furthermore, Ghazi Faisal *et al.* [17] also documented bacterial cell adhesion percentages ranging from 5.66±0.66% to 88.33±2.18% [17]. This underscores the variability in cell adherence capabilities among different bacterial isolates, emphasizing the significance of such assessments in biosurfactant production studies.

In further investigation of biosurfactant production, the 10 isolates that exhibited positive results in the screening tests were subjected to analysis of emulsification activity. The emulsification index served as an indirect technique for screening biosurfactant production, based on the assumption that the presence of biosurfactants in the cell-free culture broth would lead to emulsification of hydrocarbons [29]. Successfully, the cell-free culture broth of the 10 isolates was emulsified with petrol, kerosene and diesel, serving as hydrocarbon substrates. The emulsification index (EI24) of the study isolates ranged from 13.33±0.02% (B18) to 66.29±0.03% (B24). Similarly, Curiel-Maciel *et al.* [30] observed an emulsification index range of 19.6–63.2% in their study [30]. The highest emulsification activity was demonstrated by isolate B24 (66.3 %) on kerosene oil, followed by B22 (EI24 63.33 %), B12 (EI24 55.56%) and B20 (EI24 53.33%). Notably, the emulsification index of the isolates was significantly higher on kerosene compared to petrol and diesel oil (Table 2). Values of the emulsification index exceeding 40% are generally considered satisfactory [31]. Previous research indicates that the emulsification index value for kerosene oil typically ranges from 42% to 55% [30]. It is important to note that the stable interaction between the hydrophobic and hydrophilic phases, resulting in emulsion formation, is largely influenced by the solvents used [22,32].

**Table 2.** Emulsification activity and surface tension reduction of bacteria

Isolate no.	Emulsification index (EA%_24_)			Surface tension (mN m^−1^)			
	**Petrol**	**Kerosene**	**Diesel**	**MSM media(5 %glucose)**	**MSM(glucose+petrol oil)**	**MSM(glucose+kerosene)**	**MSM(glucose+diesel)**
Water	0	0	0	70.8±0.2	70.3±0.3	70.7±0.3	70.7±0.3
Media	0	0	0	58.3±0.8	40.5±0.3	52.3±1.4	52.3±1.4
B2	17.3±0.2	12.7±0.1	3.3±0.8	34.7±0.3	24.7±0.2	29.0±0.7	29.0±0.7
B4	35.0±0.3	5.3±0.1	3.6±0.4	33.0±0.7	26.0±0.8	25.0±0.5	25.0±0.5
B9	41.2±0.2	13.9±0.1	38.3±0.8	38.7±0.8	27.5±0.9	29.6±0.6	29.7±0.7
B12	55.6±0.3	14.8±0.2	33.7±0.8	40.0±0.7	26.6±0.2	26.5±0.7	26.5±0.7
B18	13.3±0.2	3.7±0.2	8.7±0.2	30.7±0.3	36.0±0.5	25.0±0.7	25.0±0.7
B20	53.3±0.3	12.1±0.2	38.3±0.5	35.8±0.4	33.0±0.5	32.5±0.4	32.5±0.4
B22	63.3±0.3	11.2±0.3	31.1±0.3	31.5±0.9	30.6±0.2	29.0±0.5	29.0±0.5
B23	36.7±0.2	5.0±0.5	3.4±0.04	33.3±0.2	28.7±0.8	32.3±0.2	32.3±0.2
**B24**	**66.9±0.3**	**22.2±0.5**	**40.8±0.2**	**30.7±0.6**	**31.7±0.7**	**30.3±0.8**	**30.3±0.8**
B 25	16.7±0.2	3.4±0.2	5.3±0.07	41.7±0.6	20.7±0.6	21.7±0.8	21.6±0.8

*All the vValues reported are averages of three replicates ± the standard error.

In the context of surface tension reduction, a surfactant capable of lowering water’s surface tension is referred to as an effective surfactant or biosurfactant. An effective biosurfactant achieves optimal surface activity by reducing the surface tension of water from 72 to below 35 mN m^−1^ [8]. One of the simplest screening techniques involves directly measuring the surface activity of the culture supernatant, providing a clear indication of biosurfactant production. Surface activity measurements were conducted on the culture supernatants of the 10 isolates in two distinct types of culture broths. In one broth, only 5% of the carbon source consisted of glucose, while in the other, petrol oil was added along with glucose. Notably, isolates B2, B4, B18, B22, B23 and B24 successfully reduced the surface tension of the media (MSM +5% glucose) from 58.33±0.88 to below 35 mN m^−1^ (Table 2). The biosurfactants produced by these isolates in the culture broth also demonstrated potential by effectively reducing surface tension when petrol oil and diesel oil were utilized in the media. This suggests their capability to degrade petroleum hydrocarbons, making them promising candidates for sustainable solutions in oil spill cleanup and ecosystem protection. Among the 10 isolates screened for biosurfactant production, isolate B24 stood out and was selected for further investigation. This isolate displayed the highest emulsification activity compared to the others and exhibited exceptional stability and effectiveness in reducing surface tension. Consequently, isolate B24 was identified as the most promising candidate for further exploration of its potential applications in oil recovery and environmental remediation.

### Morphological and biochemical characteristics of isolate B24

**Fig. 2.**
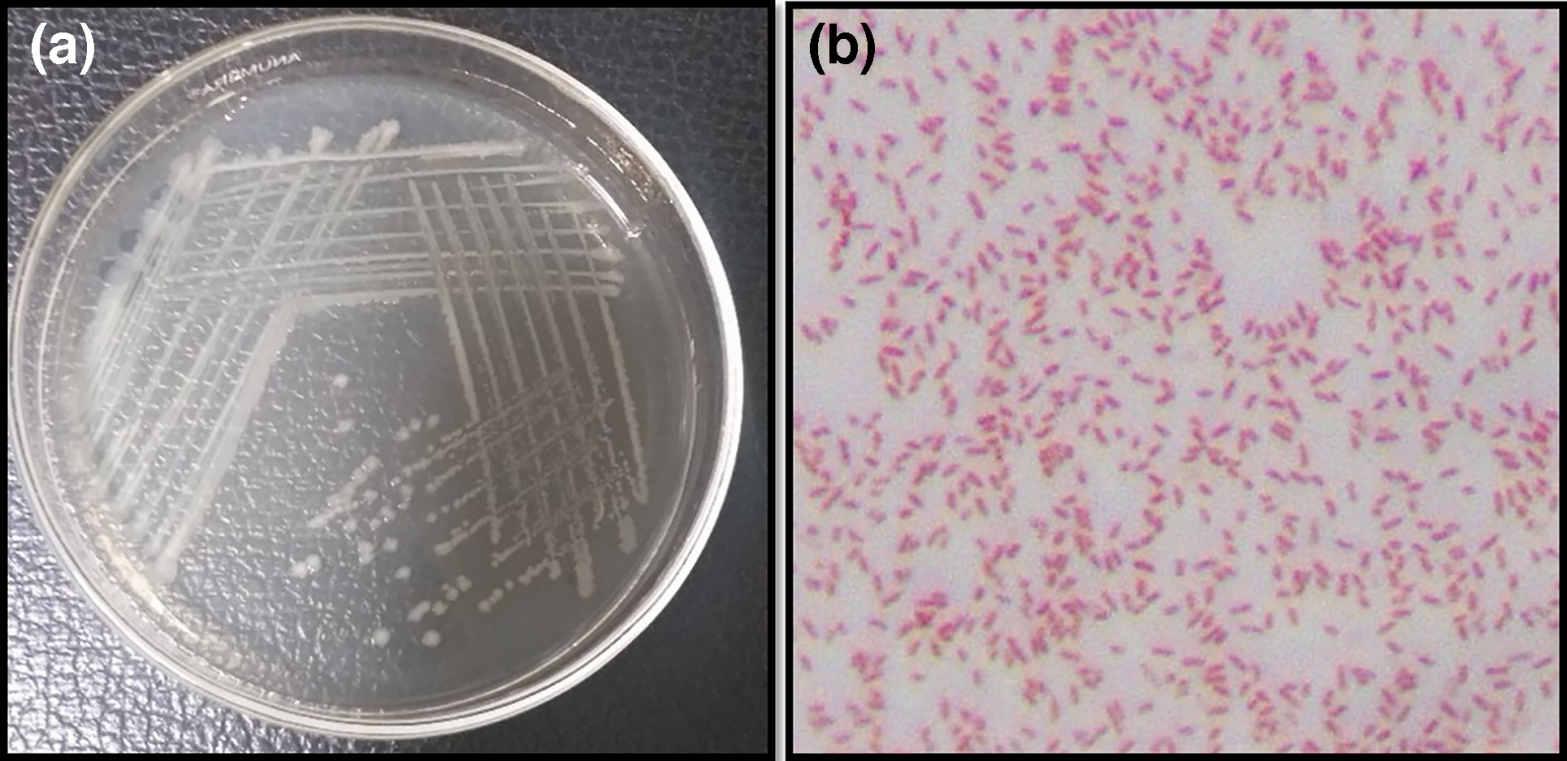
Morphological observation. (a) Bacterial growth on agar plate and (b) microscopic view.

The non-spore-forming rod exhibited positive results for the Voges–Proskauer test, citrate utilization test, catalase and motility, while showing negative results for oxidase production and the MR test (Table 3; Fig. 2).

**Table 3.** Morphological and biochemical characteristics of isolate B24

Tests	Results
Gram staining	Negative
Shape	Rod
Oxidase	Negative
Catalase	Positive
MR test	Negative
VP test	Positive
Citrate	Positive
Motility	Positive

### Molecular identification and phylogenetic analysis of isolate B24

**Fig. 3.**
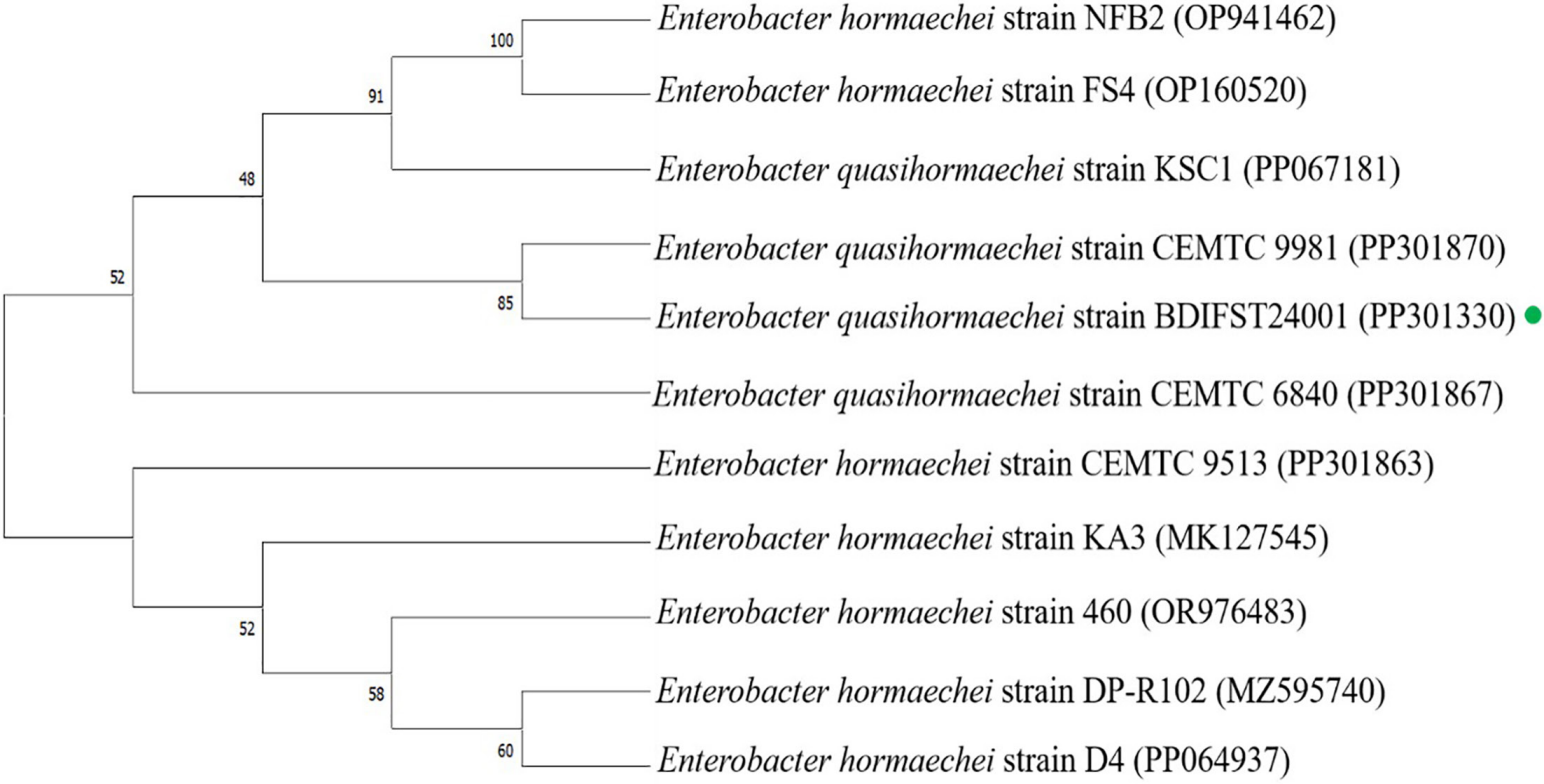
Phylogenetic analysis of *E. quasihormaechei* strain BDIFST24001 with other biosurfactant-producing bacteria calculated with the programme mega 11.0 using the neighbour-joining method.

The genomic DNA of B24 underwent extraction and confirmation using agarose gel electrophoresis. Subsequently, the 16S rRNA of the isolate was amplified and sequenced, revealing a 99.45% similarity to *E. quasihormaechei* through blast analysis. The identified gene was then submitted to GenBank, resulting in the accession number PP301330, classifying bacterial isolate B24 as *E. quasihormaechei* strain BDIFST24001. For phylogenetic analysis, reference sequences were obtained from NCBI’s GenBank sequence database, and the 16S rRNA gene sequence was aligned using the ClustalW algorithm. Analysis was conducted using mega 11 software, employing the bootstrap method with 1000 replications to evaluate the stability of the phylogenetic tree’s topology (Fig. 3). This study highlights the potential of the biosurfactant produced by * E. quasihormaechei* strain BDIFST24001 to emulsify hydrocarbons, including crude oil and other vegetable oils. Notably, there have been no previous reports of *E. quasihormaechei* strain BDIFST24001 producing biosurfactants. Described as Gram-negative *Bacilli* of the Enterobacteriaceae family by Wang *et al.* [33], *E. quasihormaechei* was isolated from a human sputum sample [33]. Morphological and biochemical tests conducted in our study align with those of previous research.

The strain effectively reduced the surface tension of the media from 58.3±0.8 to 30.7±0.6 mN m^−1^. Additionally, Rooney *et al.* [12] reported the production of rhamnolipid biosurfactant from *E. hormaechei*, achieving a similar surface tension reduction to our findings. Emulsification activities of *E. hormaechei* on various hydrocarbons and edible oils were observed in our experiment, corroborated by studies by Curiel-Maciel *et al.* [30]. It is crucial to note that while some *Enterobacter* species could be pathogenic to humans, not all strains within these species possess virulence factors necessary to cause infection. Previous studies have also identified *Enterobacter* spp. bacteria as potential candidates for microbial enhanced oil recovery [34,35].

### Fermentation and biosurfactant production

**Fig. 4.**
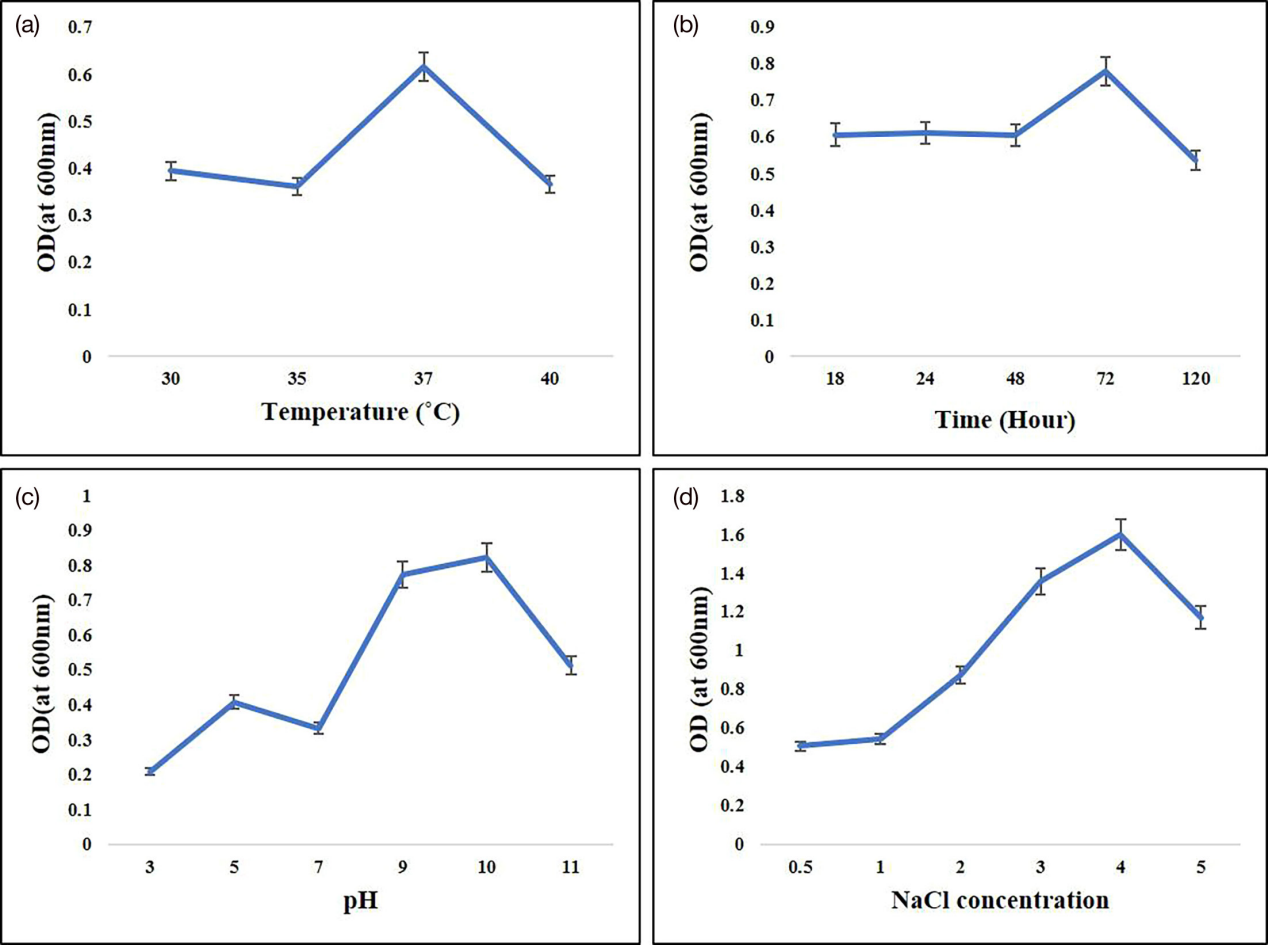
Optimization of bacterial strain. (A) Temperature. (B) incubation time. (C) pH. (D) NaCl concentration (*n*=3, mean±SD).

Selected *E. quasihormaechei* strain BDIFST24001 was cultivated in nutrient-rich media optimized for biosurfactant production. Fermentation conditions, including temperature, time period, pH and NaCl concentration were optimized to maximize biosurfactant yield (Fig. 4). For optimization purposes, the temperature was varied between 30 and 40 °C, with the highest growth observed at 37 °C covering a 72-h period. To optimize pH conditions, a range of 3, 5, 7, 9, 10 and 11 was explored. Growth rates were monitored at different NaCl concentrations (0.5, 1, 2, 3 and 4 %). Following experimentation, the optimal pH for the growth of *E. quasihormaechei* strain BDIFST24001 and biosurfactant production was determined to be 10.0. This pH level provided the most favourable conditions for bacterial growth and subsequent biosurfactant yield. Similarly, the optimal temperature for maximum biosurfactant production was identified as 37 °C, ensuring the highest output. Furthermore, the investigation revealed that a high salinity concentration of 4% was most conducive for both production and extraction processes. This elevated salinity level was found to provide essential ions necessary for bacterial growth and efficient biosurfactant production. These findings underscore the significance of optimizing pH, temperature and salinity parameters in the culture media to enhance the growth of *E. quasihormaechei* strain BDIFST24001 and maximize biosurfactant production.

### Purification of biosurfactant

The purification of biosurfactants from crude fermentation broths of *E. quasihormaechei* strain BDIFST24001 is a crucial step to obtain high-purity compounds suitable for applications. Several techniques are employed for biosurfactant purification, including solvent extraction, precipitation and chromatography. Solvent extraction using organic solvents like chloroform and methanol is a commonly utilized method to separate biosurfactants from the aqueous phase. This technique effectively isolates biosurfactants into the organic solvent layer, leaving behind impurities in the aqueous phase. Precipitation methods, including acid precipitation, are also employed to concentrate biosurfactants by selectively precipitating impurities while retaining the biosurfactant in solution. This technique aids in the removal of unwanted substances, resulting in a more purified biosurfactant product. Chromatographic techniques, such as silica gel column chromatography, are utilized for further purification and separation of biosurfactant fractions based on their chemical properties. This method allows for the isolation of specific components within the biosurfactant mixture, enhancing purity. After purification, the yield of biosurfactant obtained was determined to be 430 mg l^−1^. This high yield suggests the effectiveness of the purification techniques used, resulting in a purified biosurfactant product suitable for various commercial applications.

### Characterization of biosurfactant

Characterization of biosurfactant is essential to understand its chemical structure, physicochemical properties and functional characteristics. Various analytical techniques are employed for the characterization of biosurfactant, including spectroscopic methods, chromatographic techniques, UV-vis spectroscopy, surface tension measurements and mass spectrometry. Fourier-transform infrared (FTIR) spectroscopy is commonly used for structural elucidation of biosurfactants, providing information about their chemical composition, functional groups and molecular conformation. Gas chromatography (GC)-FID is valuable for identifying the fatty acid or amino acid moieties present in biosurfactant molecules. Surface tension measurement using tensiometers is employed to determine the surface-active properties of biosurfactants, including critical micelle concentration (CMC), surface tension reduction and emulsification index. Furthermore, assessing interfacial tension between oil and water phases offers valuable insights into the efficacy of biosurfactants in improving oil recovery and emulsification.

**Fig. 5.**
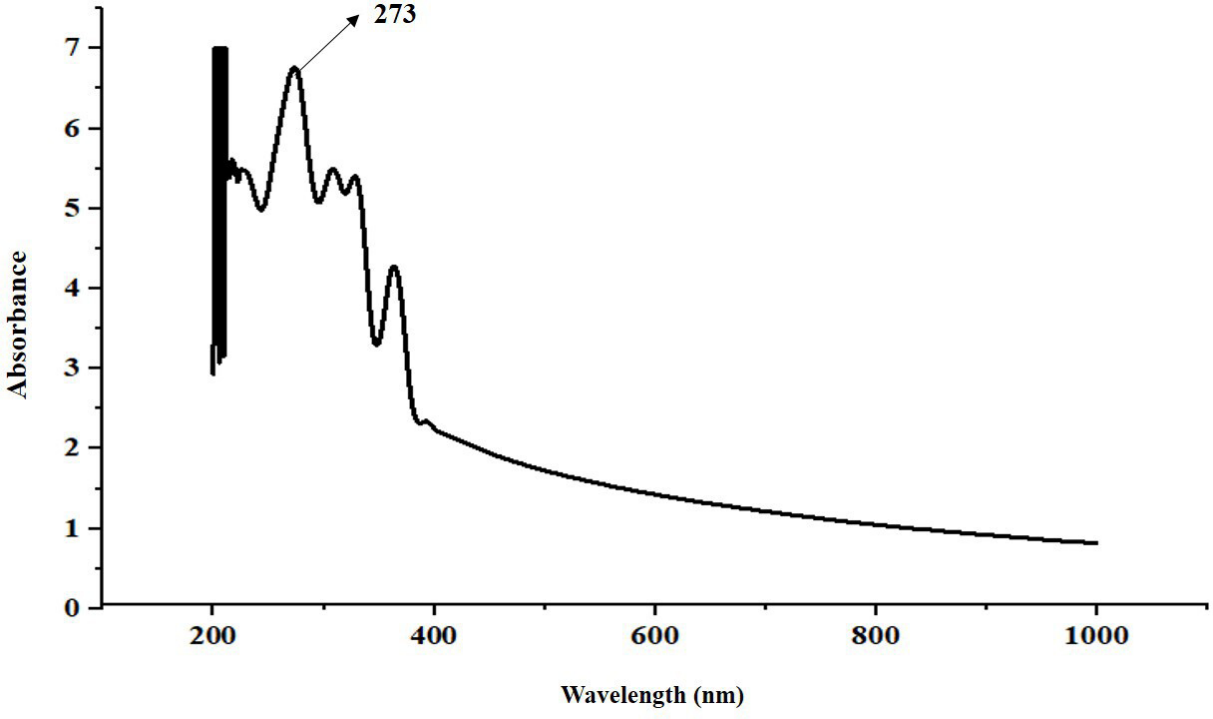
UV-visible spectrum of the biosurfactant.

UV-visible spectroscopy aids in identifying functional groups based on their characteristic absorption bands. In the extracted biosurfactant from *E. quasihormaechei* strain BDIFST24001, a maximum peak was observed at 273 nm (Fig. 5) (Data S1, available in the online version of this article). This finding aligns with previous research by Magthalin *et al.* [36], who also observed a peak at 264 nm for glycolipid biosurfactants [36]. Glycolipid biosurfactants have garnered significant interest for biotechnological applications. Usually consisting of a carbohydrate moiety and a fatty acid connected through a glycosidic bond, glycolipids provide biodegradable surface-active characteristics, presenting potential as eco-friendly substitutes for synthetic emulsifiers. This characteristic UV-visible spectrum provides valuable insight into the molecular composition of the biosurfactant produced by * E. quasihormaechei* strain BDIFST24001, further supporting its potential for various biotechnological applications, including oil recovery enhancement and environmental remediation.

**Fig. 6.**
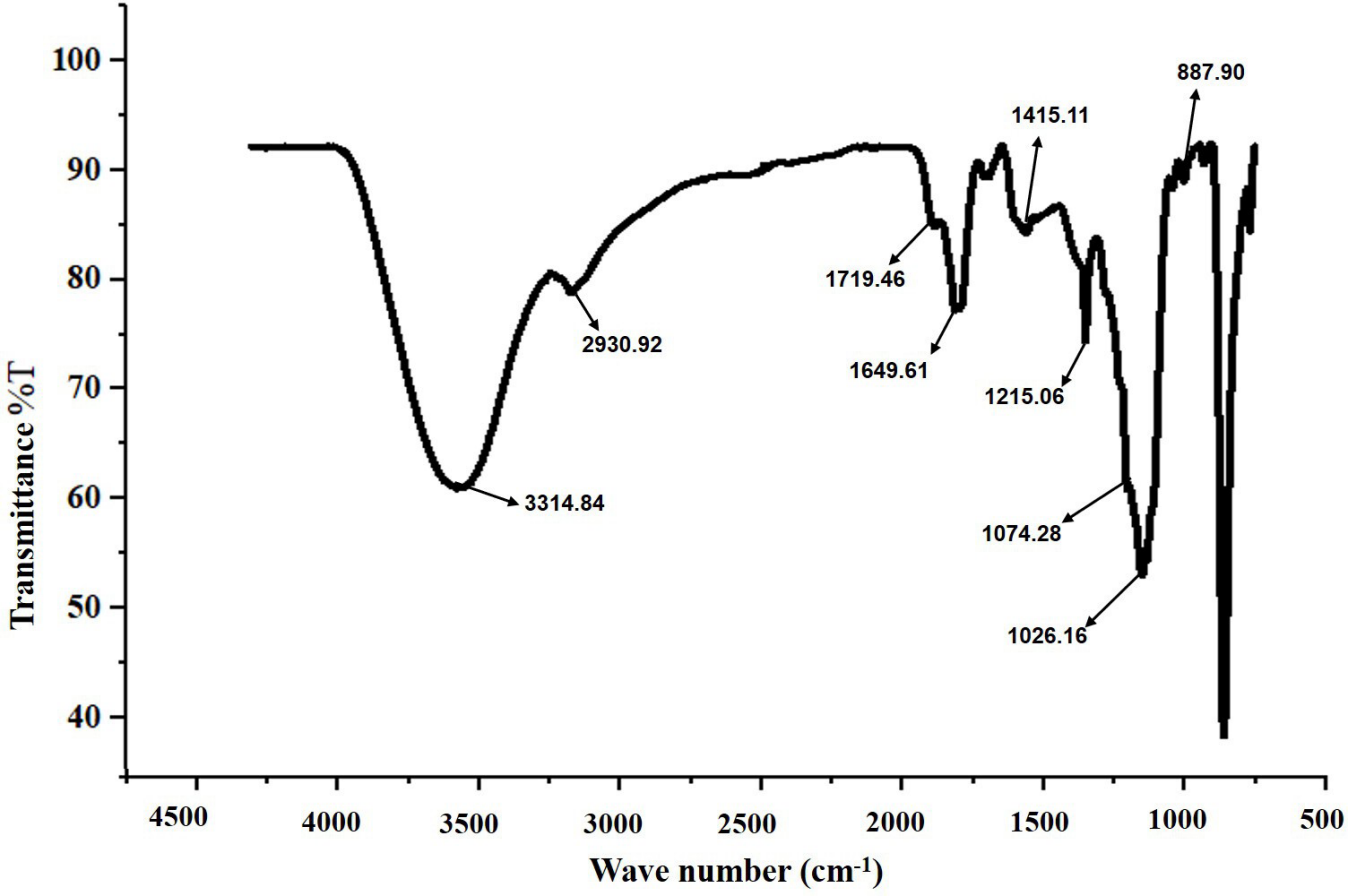
FT-IR of biosurfactant produced by *E. quasihormaechei* strain BDIFST24001.

The FT-IR spectra of the isolated and purified biosurfactants revealed characteristic absorbance peaks indicative of glycolipid structure. FT-IR analysis confirmed the presence of a polysaccharide moiety and aliphatic hydrocarbon chains (lipid), supporting the glycolipid form of the biosurfactant. Specific functional groups were identified through characteristic absorbance peaks: the long, sharp band at 3314.84 cm^−1^ indicated hydroxyl (–OH) groups, while bands at 2930.92 cm^−1^ represented alkyl groups (C–H) of alkanes. Peaks at 1719.46 cm^−1^ revealed a carbonyl group (C=O) conjugated with sugar groups, while a peak at 1074 cm^−1^ corresponded to the C–H stretch bond in aliphatic amines. Absorption peaks at 1451.11 and 1215.06 cm^−1^ illustrated carbon atom stretching in the sugar moiety, with additional peaks at 1074.28 and 887.90 cm^−1^ linked to glycosidic linkage stretching vibrations, confirming the glycolipid nature (Fig. 6). These findings were consistent with previous studies of the glycolipid characterization [22,23, 37].

Similarly, the biosurfactant (BS) exhibited a broad peak at 3336 cm^−1^ characteristics of the –OH functional group due to hydrogen bonding, indicating the presence of polysaccharides. Sharp bands at 2856 and 2924 cm^−1^ indicated C–H bands (CH_2_–CH_3_) in the hydrocarbon chain, while absorption around 1738 cm^−1^ and a weak peak at 1456 cm^−1^ represented ester carbonyl groups (CO in COOH). A stretching peak observed at 1645 cm^−1^ was related to ester compounds, while absorption around 1051 cm^−1^ was assigned to C–O–C in the rhamnose molecule in the BS. The absence of bands around 1550, 3420 and 3245 cm^−1^ confirmed the absence of N–H bonds, further confirming the absence of amino acid characteristic of lipopeptide-type BS. Together, these results supported the conclusion that the BS had a rhamnolipid structure with rhamnose rings and long hydrocarbon chains.

The FT-IR analysis further suggested that the biosurfactant was likely glycolipid in nature, as characteristic absorption bands corresponding to functional groups of glycolipids were observed. These bands included 3340 cm^−1^, O–H stretching, stretching bands of amide and ester C=O groups at approximately 1637 and 1100 cm^−1^ and N–H and C–N–H vibrations at around 1637 and 600 cm^−1^, respectively. These observations, coupled with the absence of amino acids and the presence of carbohydrate and lipid compounds, supported the inference that the biosurfactant belonged to the glycolipid category. In contrast, the biosurfactant component of *Azotobacter chroococcum* was identified as a lipopeptide, with FT-IR analysis revealing characteristic wave numbers indicative of aliphatic chain presence. Additionally, the presence of N–H and CO–N bonds, along with observed C–O bonds, supported the identification of lipopeptide moieties. Overall, the FT-IR analysis provided valuable insights into the chemical composition and structure of the studied biosurfactants, supporting their characterization as rhamnolipids (Data S2).

The fatty acid composition of the purified biosurfactant was determined through GC-FID analysis, revealing that the biosurfactant primarily consisted of long-chain fatty acids, predominantly C-16 long-chain fatty acids. Specifically, the major fatty acid identified in the biosurfactant was C-16 hexadecanoic fatty acid, also known as palmitic acid. This finding is consistent with previous studies on glycolipids, where hexadecanoic acid was frequently identified as a major fatty acid chain associated with the biosurfactant. In addition to hexadecanoic acid, the current study identified new fatty acids produced by *E. quasihormaechei* strain BDIFST24001, including lauric acid (C12 : 0), eicosenoic acid (C20 : 1) and behenic acid (C22 : 1). Furthermore, palmitic acid, myristic acid and stearic acid contents were also detected in the biosurfactant (Data S3). These findings expand the understanding of the fatty acid composition of biosurfactants produced by *E. quasihormaechei* strain BDIFST24001, indicating a diverse range of fatty acid chains present. Comparisons with other studies further support the diversity of fatty acids found in biosurfactants. For instance, previous research on biosurfactants extracted from *Lactobacillus lactis* identified octadecanoic acid as a fatty acid chain associated with the sugar moiety, in addition to palmitic acid. Moreover, rhamnolipids, another type of glycolipid, commonly include β-hydroxydecanoic acid molecules as branched fatty acids, adding to the variety of fatty acids observed in biosurfactants. Similarly, studies on biosurfactants produced by other bacteria, such as *Lactobacillus pentosus*, have highlighted palmitic acid and stearic acid as major fatty acids. The fatty acid composition of the biosurfactant in the present study, including palmitic acid, myristic acid and stearic acid, aligns with these previous findings. Overall, the fatty acid analysis presented in Table 4 provides insight into the composition of the biosurfactant produced by *E. quasihormaechei* strain BDIFST24001, showcasing a diverse array of fatty acid chains that contribute to its surfactant properties.

**Table 4.** Fatty acid composition of the biosurfactants

Name of fatty acid	Carbon no.	Purified biosurfactant
**Saturated**		
Capric acid	C10 : 0	0.741344
Undecanoic acid	C11 : 0	0.295252
Lauric acid	C12 : 0	0.300049
Tridecanoic acid	C13 : 0	0.482582
Myristic acid	C14 : 0	3.114734
Palmitic acid	C16 : 0	31.4864
Heptadecanoic acid	C17 : 0	0.327748
Stearic acid	C18 : 0	6.803585
Eicosenoic acid	C20 : 0	0.38186
**Monounsaturated**		
Myristoleic acid	C14 : 1	3.492859
Palmitoleic acid	C16 : 1	0.911153
Oleic acid	C18 : 1 c9	31.52749
Vaccenic acid	C18 : 1 c11	0.49
**Polyunsaturated**		
Linoleic acid	C18 : 2 c	9.826876
Linolenic acid	C18 : 3	1.347508
Total		91.52945
Unknown		8.470555

All value was expressed as percentage (%).

**Fig. 7.**
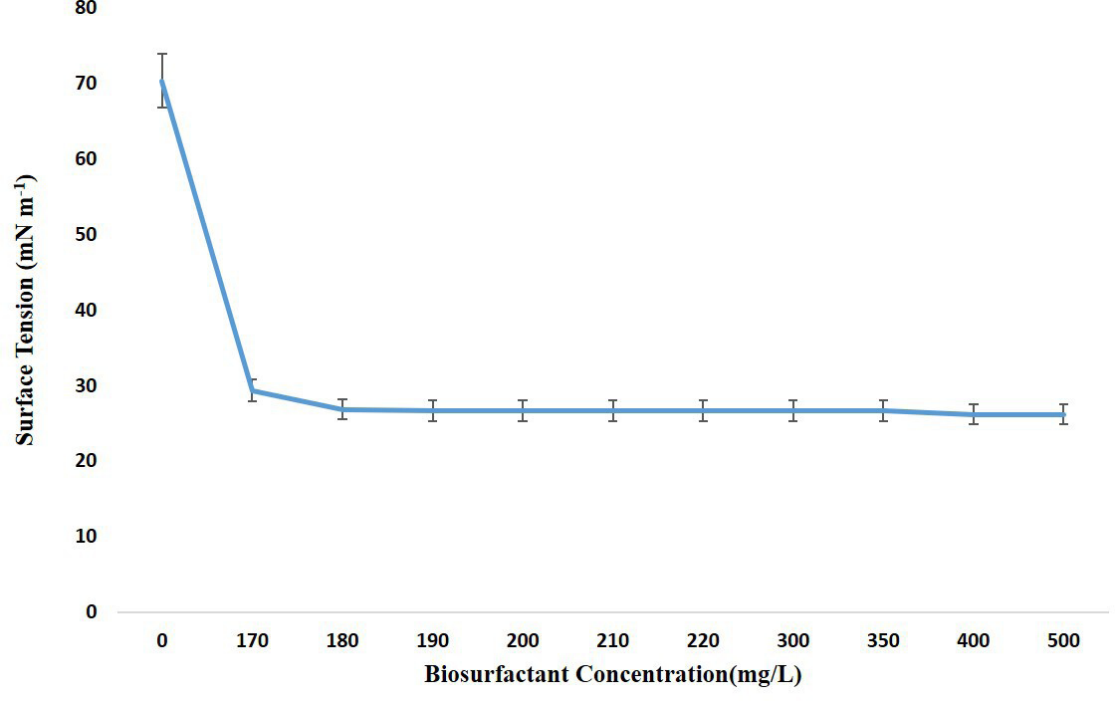
Critical micelle concentration (CMC) of the biosurfactant (*n*=3, mean±SD).

The CMC of a biosurfactant is a crucial parameter that indicates the effectiveness of the biosurfactant in reducing the surface tension of a liquid. In our study, the CMC was determined using the cell-free crude biosurfactant produced by *E. quasihormaechei* strain BDIFST24001. The surface tension of water dropped from 70.33±0.33 mN m^−1^ to <40 mN m^−1^ with the concentration of the crude biosurfactant at 120 mg l^−1^. The CMC for the extracted biosurfactant was determined to be 180 mg l^−1^, associated with a surface tension of 26.83±0.44 mN m^−1^ (Fig. 7). This result underscores the potency of the biosurfactant produced by *E. quasihormaechei* strain BDIFST24001 in reducing the surface tension of water, as concentrations as low as 120 mg l^−1^ were sufficient to achieve significant surface tension reduction. Additionally, the determined CMC value aligns with previous studies, which have reported CMC values ranging from 1 to 200 mg l^−1^ for achieving surface tension reduction below 40 mN m^−1^. Overall, the measured CMC highlights the efficacy of the extracted biosurfactant and its potential for various applications, including enhanced oil recovery, environmental remediation and industrial processes requiring surface tension reduction (Data S4).

#### Utilization of rhamnolipids for oil spill remediation

**Fig. 8.**
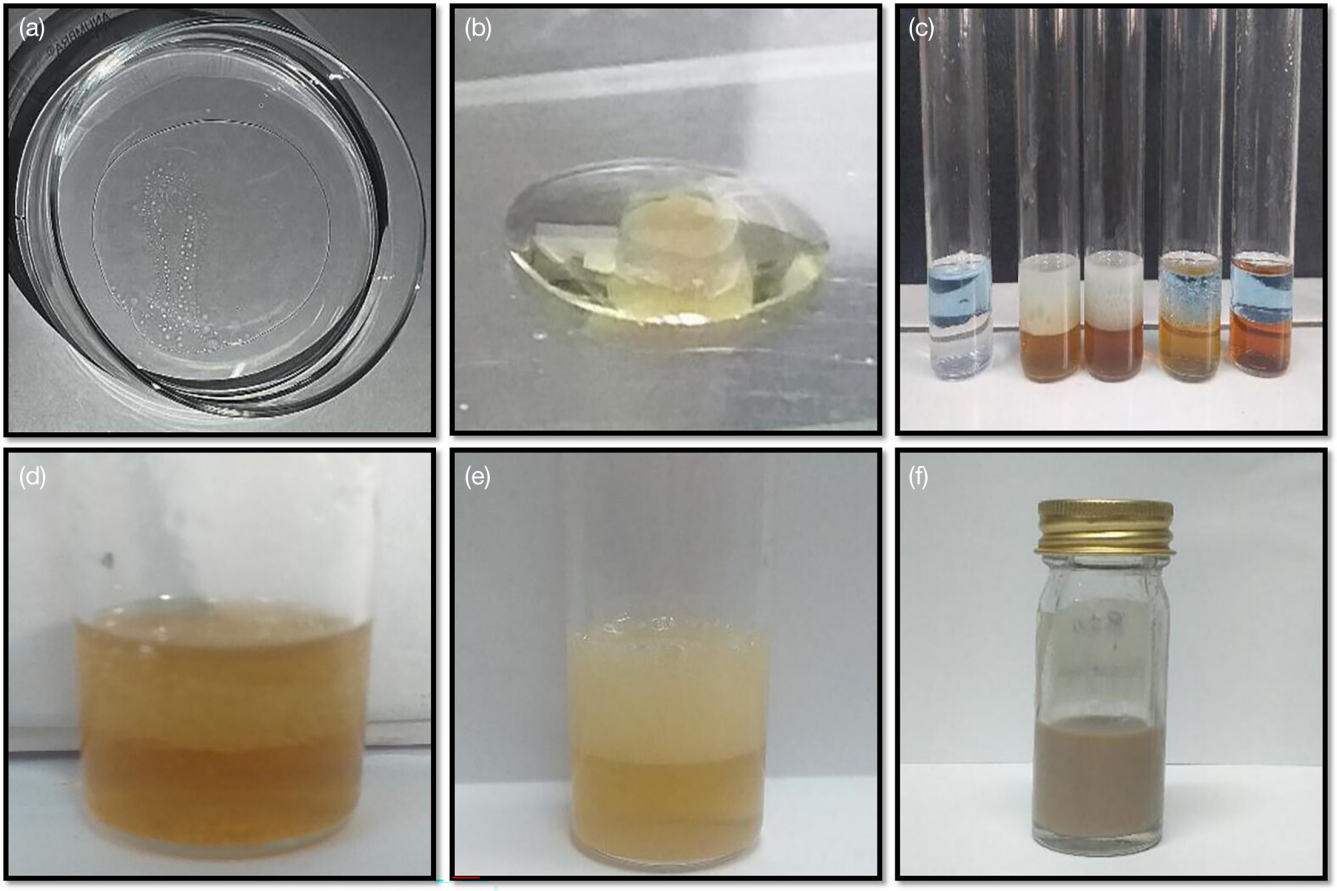
Screening of biosurfactant producing bacteria; (A) oil spreading test, (B) drop-collapse test, (C) emulsification activity (with kerosene oil), (D) emulsification activity (with diesel oil), (E) emulsification activity (with petrol oil) and (F) extraction of crude biosurfactant.

Biosurfactants have emerged as promising agents for addressing oil spill cleanup and remediation challenges due to their ability to enhance the solubilization, dispersion and emulsification of hydrophobic contaminants. Their unique amphiphilic properties allow them to interact effectively with both oil and water phases, facilitating the formation of emulsions that can be readily degraded by micro-organisms. In our study, we evaluated the efficacy of crude biosurfactant, derived from biosurfactant-producing bacteria, in removing oil spills from water, utilizing petrol oil as the contaminant (Fig. 8). Through our experimentation, we observed that the rhamnolipids effectively dispersed the oil in water, significantly aiding in its cleanup. This was attributed to the rhamnolipid’s ability to reduce the surface tension of the oil, thereby facilitating its mixing with water. These promising findings underscore the potential of rhamnolipids as valuable tools in mitigating the environmental impact of oil spills. It is noteworthy that our study represents the first investigation into rhamnolipid production by *E. quasihormaechei* strain BDIFST24001, highlighting the potential of this bacterium in rhamnolipid production. By delving deeper into the capabilities of rhamnolipids and refining our understanding of their properties, we can harness their potential to effectively address environmental challenges (Data S4).

#### Mechanism of action for oil spill remediation by rhamnolipids

**Fig. 9.**
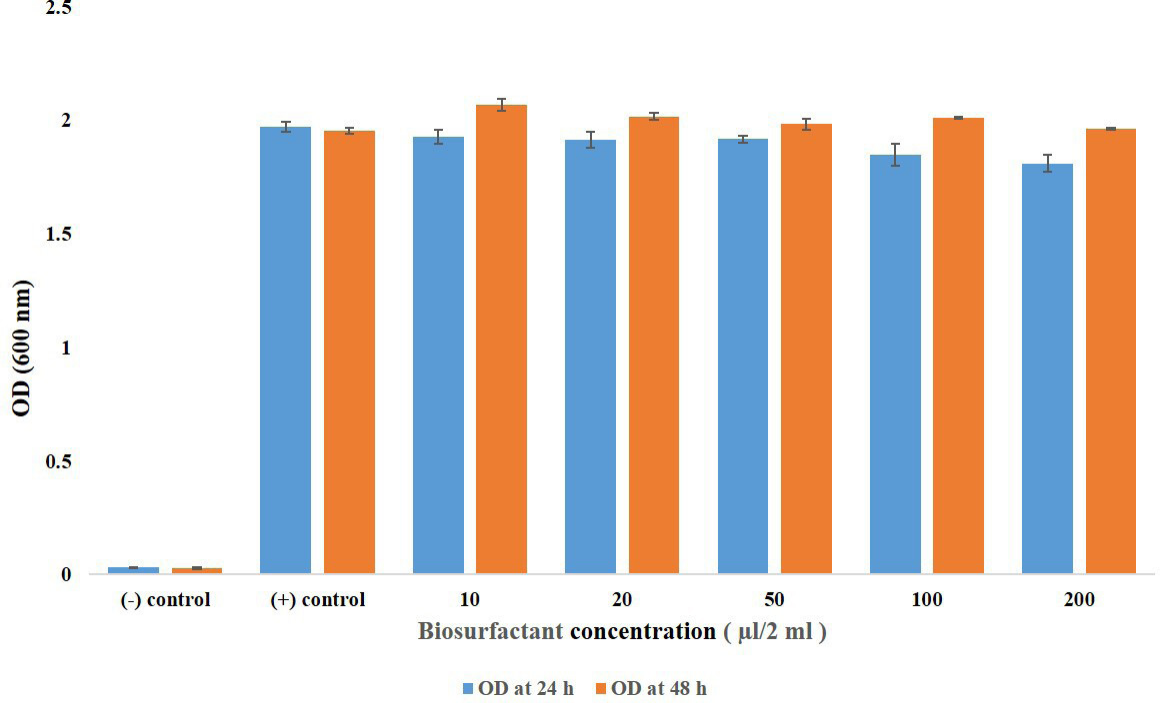
The mechanism of action of *E. quasihormaechei* strain BDIFST24001 at different concentrations of rhamnolipids varies (*n*=3, mean±SD).

Rhamnolipids possess amphiphilic properties, with both hydrophilic and hydrophobic regions. When introduced to an oil-water interface, rhamnolipids adsorb to oil droplet surfaces. Their hydrophobic tails interact with oil molecules while their hydrophilic heads face outward towards water, forming stable oil-in-water emulsions. This process disperses oil droplets throughout the water column, increasing their surface area and accessibility for microbial degradation. Rhamnolipids also reduce surface tension at the oil-water interface, aiding in dispersing oil slicks and promoting oil spread across water surfaces, thereby enhancing remediation efficiency. This property facilitates hydrocarbon accessibility to indigenous bacteria like *E. quasihormaechei* strain BDIFST24001, which metabolizes oil as a carbon source for biodegradation. Rhamnolipids do not exhibit self-toxicity towards *E. quasihormaechei* strain BDIFST24001, making them effective aids in oil spill recovery through emulsification, dispersion and enhanced biodegradation processes (Fig. 9). Additionally, a study found that zincmethylphyrins and coproporphyrins, growth stimulants released by *Sphingopyxis* sp., enabled successful laboratory cultivation of previously uncultured *Leucobacter* sp., albeit with environmental self-toxicity, indicating bidirectional functionality for these compounds [38]. *E. quasihormaechei* strain BDIFST24001 that secretes rhamnolipids benefits from conditions that support their growth, and the concentration of rhamnolipids enhances oil degradation in contaminated sites. As these bacteria multiply, they produce more rhamnolipids, which act as biosurfactants. These molecules reduce the surface tension between oil and water, facilitating the formation of emulsions that increase the surface area accessible for microbial action. This promotes a more efficient breakdown of oil into smaller components that can be further metabolized by *E. quasihormaechei* strain BDIFST24001 (Data S4). Crucially, rhamnolipids do not inhibit bacterial growth themselves, making this process advantageous for sustained oil remediation in environments affected by contamination. In summary, the conclusion would be based on rigorous experimental design, quantitative measurements of bacterial activity and statistical analysis to ensure robustness and reliability of the findings.

#### Future perspectives and challenges

Despite significant advancements in the isolation, purification and characterization of rhamnolipids from *E. quasihormaechei* strain BDIFST24001, several challenges remain to be addressed for their widespread commercialization and application in oil remediation. Optimization of fermentation conditions, scalability of production processes and cost-effectiveness are critical factors that need to be considered for industrial-scale rhamnolipid production. Furthermore, there is a need for additional research to investigate the variety of bacteria capable of producing rhamnolipids or other secondary metabolites across diverse environmental settings [39,41]. Additionally, efforts should be directed towards the development of microbial strains engineered to exhibit improved biosurfactant production capacities and other desirable properties.

In conclusion, rhamnolipids derived from bacteria hold immense potential for oil spill cleanup and environmental remediation due to their biodegradability, low toxicity and effectiveness in enhancing oil recovery processes. Continued research efforts aimed at improving rhamnolipid production, purification and application strategies will contribute to their sustainable utilization in mitigating the environmental impacts of oil pollution. Overall, the production and characterization of biosurfactants from * E. quasihormaechei* strain BDIFST24001 offer a sustainable and effective solution for addressing oil spill contamination, underscoring the importance of further research and development in this field to maximize their potential benefits for environmental protection and restoration.

## supplementary material

10.1099/acmi.0.000830.v4 Uncited Supplementary Data Sheet 1.

10.1099/acmi.0.000830.v4 Uncited Supplementary Data Sheet 2.

10.1099/acmi.0.000830.v4 Uncited Supplementary Data Sheet 3.

10.1099/acmi.0.000830.v4 Uncited Supplementary Data Sheet 4.

## Abbreviations

BATH: bacterial adhesion to hydrocarbon
CMC: critical micelle concentration
IMViC: indole, methyl red, Voges Proskauer and citrate
ITOPF: International Tanker Owners Federation
LB: Luria-Bertani
LMW: low-molecular-weight
MR: methyl red
MSM: minimal salt medium
OD: optical density
SDS: sodium dodecyl sulphate
VP: Voges-Proskauer
